# Aerobic exercise suppresses CCN2 secretion from senescent muscle stem cells and boosts muscle regeneration in aged mice

**DOI:** 10.1002/jcsm.13526

**Published:** 2024-06-26

**Authors:** Fan Li, Fulong Zhang, Haiwang Shi, Honglin Xia, Xiaobei Wei, Siqi Liu, Tao Wu, Yuecheng Li, Feng Shu, Mengjie Chen, Jie Li, Rui Duan

**Affiliations:** ^1^ School of Physical Education and Sports Science South China Normal University Guangzhou 510006 China; ^2^ School of Physical Education Shanxi Datong University Datong China

**Keywords:** aerobic exercise, aging, CCN2, fibrosis, MuSCs, skeletal muscle

## Abstract

**Background:**

Aging negatively impacts tissue repair, particularly in skeletal muscle, where the regenerative capacity of muscle stem cells (MuSCs) diminishes with age. Although aerobic exercise is known to attenuate skeletal muscle atrophy, its specific impact on the regenerative and repair capacity of MuSCs remains unclear.

**Methods:**

Mice underwent moderate‐intensity continuous training (MICT) from 9 months (aged + Ex‐9M) or 20 months (aged + Ex‐20M) to 25 months, with age‐matched (aged) and adult controls. Histological examinations and MuSC transplantation assays assessed aerobic exercise effects on MuSC function and muscle regeneration. CCN2/connective tissue growth factor modulation (overexpression and knockdown) in MuSCs and AICAR supplementation effects were explored.

**Results:**

Aged mice displayed significantly reduced running duration (65.33 ± 4.32 vs. 161.9 ± 1.29 min, mean ± SD, *P* < 0.001) and distance (659.17 ± 103.64 vs. 3058.28 ± 46.26 m, *P* < 0.001) compared with adults. This reduction was accompanied by skeletal muscle weight loss and decreased myofiber cross‐sectional area (CSA). However, MICT initiated at 9 or 20 months led to a marked increase in running duration (142.75 ± 3.14 and 133.86 ± 20.47 min, respectively, *P* < 0.001 compared with aged mice) and distance (2347.58 ± 145.11 and 2263 ± 643.87 m, respectively, *P* < 0.001). Additionally, MICT resulted in increased skeletal muscle weight and enhanced CSA. In a muscle injury model, aged mice exhibited fewer central nuclear fibres (CNFs; 266.35 ± 68.66/mm^2^), while adult, aged + Ex‐9M and aged + Ex‐20M groups showed significantly higher CNF counts (610.82 ± 46.76, 513.42 ± 47.19 and 548.29 ± 71.82/mm^2^, respectively; *P* < 0.001 compared with aged mice). MuSCs isolated from aged mice displayed increased CCN2 expression, which was effectively suppressed by MICT. Transplantation of MuSCs overexpressing CCN2 (Lenti‐CCN2, Lenti‐CON as control) into injured tibialis anterior muscle compromised regeneration capacity, resulting in significantly fewer CNFs in the Lenti‐CCN2 group compared with Lenti‐CON (488.07 ± 27.63 vs. 173.99 ± 14.28/mm^2^, *P* < 0.001) at 7 days post‐injury (dpi). Conversely, knockdown of CCN2 (Lenti‐CCN2shR, Lenti‐NegsiR as control) in aged MuSCs improved regeneration capacity, significantly increasing the CNF count from 254.5 ± 26.36 to 560.39 ± 48.71/mm^2^. Lenti‐CCN2 MuSCs also increased fibroblast proliferation and exacerbated skeletal muscle fibrosis, while knockdown of CCN2 in aged MuSCs mitigated this pattern. AICAR supplementation, mimicking exercise, replicated the beneficial effects of aerobic exercise by mitigating muscle weight decline, enhancing satellite cell activity and reducing fibrosis.

**Conclusions:**

Aerobic exercise effectively reverses the decline in endurance capacity and mitigates muscle atrophy in aged mice. It inhibits CCN2 secretion from senescent MuSCs, thereby enhancing skeletal muscle regeneration and preventing fibrosis in aged mice. AICAR supplementation mimics the beneficial effects of aerobic exercise.

## Introduction

One of the hallmarks of mammalian aging is the diminished regenerative potential of tissues.^1^ Skeletal muscle relies on muscle stem cells (MuSCs), or satellite cells, for regeneration. Positioned between the sarcolemma and basal lamina of muscle fibres, MuSCs are activated during postnatal myogenesis through myo‐trauma, exercise or stress, leading to the formation of myoblasts. These myoblasts proliferate, differentiate and either fuse with existing myofibers or give rise to new myofibers. However, in aging individuals, dysregulated autophagy causes MuSCs to enter senescence due to the loss of proteostasis.^2^ Overactivation of the P38 α/β mitogen‐activated protein kinase (MAPK) pathway increases asymmetric division, resulting in elevated myogenic progenitor cell production, decreased MuSC production and impaired self‐renewal.^3^, ^4^ Impaired Notch signalling leads to reduced p53 activity and mitotic catastrophe in aged MuSCs.^5^ In geriatric mice, resting MuSCs switch from a reversible quiescent state to an irreversible presenescent state due to derepression of p16(INK4a).^6^ The aged MuSC niche also impairs MuSC function, with increased interleukin 6 (IL6) levels leading to the overactivation of the signal transducer and activator of transcription 3 (STAT3) signalling pathway in MuSCs, promoting asymmetric division of MuSCs.^7^ Upregulated expression of fibroblast growth factor 2 (FGF2) in muscle fibres results in the loss of quiescence and depletion of MuSCs.^8^ The decline in interferon‐gamma (IFN‐γ) response macrophage proportion,^9^ inefficient glutamine secretion of macrophages^10^ and impaired matricellular WISP1 (NT1‐inducible signalling pathway protein 1 secretion from fibro‐adipogenic progenitors [FAPs])^11^ contribute to MuSC dysfunction in aged mice, impairing skeletal muscle regeneration. Notably, exposure of MuSCs from old mice to a young environment by heterochronic parabiosis restored the activation of Notch signalling as well as the proliferation and regenerative capacity of aged satellite cells.^12^

In 2007, Andrew S. Brack and his colleagues found the MuSCs from aged mice tend to convert from a myogenic to a fibrogenic lineage due to the activation of the canonical Wnt signalling pathway, impairing muscle regeneration and enhancing the fibrotic response.^13^ Muscular fibrosis profoundly impacts muscular function and has been linked to sarcopenia and muscular dystrophies.^14^ However, in aged mice^13^ and Mdx mice,^15^ MuSCs also serve as precursors for myofibroblasts. The myogenic‐to‐fibrogenic conversion of MuSCs impaired myogenic ability and also caused skeletal muscle fibrosis. Exercise, recognized as a strategy to enhance well‐being and counteract aging, has been substantiated to ameliorate the regenerative reparative potential in aging mice by reinstating cyclin D1.^16^ Whether exercise can alleviate the conversion of MuSCs from a myogenic to a fibrogenic lineage is still unknown. Further study has shown that MuSCs regulate collagen biosynthesis in fibroblasts through secreted exosomes containing miR‐206 in response to hypertrophic stimulation.^17^ Aging induces an increase in skeletal muscle fibrosis before and after injury.^11^ Although evidence exists for MuSC‐mediated regulation of ECM (extracellular matrix) production during hypertrophic stimulation, the role of activated satellite cells in the modulation of their extracellular niche during aging is largely unexplored.

This study aimed to investigate the impact of aging on MuSCs and its subsequent role in triggering skeletal muscle fibrosis and to explore the potential influence of exercise on these processes. Our findings reveal that aging contributes to the development of skeletal muscle fibrosis, and the secretions from MuSCs derived from aged mice enhance the proliferation of fibroblasts. Through RNA‐sequencing (RNA‐seq) analysis, we identified an upregulation of CCN2 expression in senescent MuSCs, and subsequent overexpression of CCN2 in young MuSCs was observed to stimulate fibroblast proliferation both in vitro and in vivo. Notably, the implementation of exercise or AICAR administration enhanced skeletal muscle regenerative capabilities while concurrently mitigating the progression of skeletal muscle fibrosis.

## Methods

### Animals

The study received approval from the Ethics Committee of the South China Normal University School of Sports Science. Male C57BL/6J mice (5–8 months old) were obtained from Southern Medical University. Various mouse strains, including Pax7CreER, R26R‐EYFP, ROSA26DTA and Mdx, were procured from The Jackson Laboratory. Mice were housed in specific‐pathogen‐free conditions with a 12‐h light–dark cycle and ad libitum access to food.

### Exercise

In the normal training session, male C57BL/6 mice were individually housed and subjected to moderate‐intensity aerobic exercise on a treadmill. The training protocol included a 5‐min warm‐up at 5 m/min, followed by a 42‐min run at 60% of their maximum running speed and a 5‐min cool‐down at 5 m/min every 2 days. Two groups of mice commenced exercise at either 9 months (aged + Ex‐9M) or 20 months (aged + Ex‐20M) of age, continuing until 25 months of age.

Maximum running speed was determined through exhaustion tests conducted monthly to adjust exercise intensity. During these tests, mice began exercising at 10 m/min, with speed increasing by 3 m/min every 3 min until exhaustion. The maximum running speed was determined by subtracting 3 m/min from the speed at exhaustion. Normal training was skipped during the exhaustion test. This training protocol and exercise intensity adjustment methods were also applied to the MICT‐10W group, conducted from 22.5 to 25 months old, lasting for 10 weeks.

### Endurance running test

Mice underwent an endurance running test starting at 10 m/min. After 40 min, the treadmill speed increased by 1 m/min every 10 min for the next 30 min. Subsequently, the speed increased by 1 m/min every 5 min until the mice reached a point of exhaustion.^18^ This test was conducted 2 days after completing either the 9‐ or 20‐month normal training procedures.

### Mice AICAR administration

Mice received a subcutaneous injection of 250 mg/kg AICAR (Selleck, #s1802) or saline every other day.

### Muscle injury

Mice were anaesthetized with Avertin (250 mg/kg). Muscle injury was induced using 10 μM of CTX (cardiotoxin; 50 μL) or 1.2% barium chloride (Macklin, #B861682‐25g) for regeneration assays.

### Muscle stem cell isolation

Mice were anaesthetized using avodin (250 mg/kg) and underwent cold physiological saline perfusion. Limbs were treated with 70% ethanol, and subsequently, quadriceps femoris, triceps calf and tibialis anterior (TA) muscles were carefully extracted and placed in sterile, cold phosphate‐buffered saline (PBS). The skeletal muscles underwent a series of washes, initially with 75% ethanol, followed by cold PBS. The skeletal muscle was then mechanically disrupted into pieces for a few minutes in a 60‐mm petri dish using sterile scissors. For enzymatic digestion, approximately 2 mL of collagenase/dispase/CaCl_2_ solution (consisting of 1.5 U/mL of collagenase D [Roche, #11088858001], 2.4 U/mL of disperse enzyme II [Sigma, #D4693‐1g] and 2.5 mM of CaCl_2_) was added per gram of tissue. This mixture was incubated at 37°C for ~1 h. During the incubation, pipetting up and down occurred at 30‐, 40‐ and 50‐min intervals using a Pasteur pipette. This process continued until the tissue mixture turned into a fine slurry. Following incubation, the digestion was halted by adding 10 mL of F10 muscle satellite cell culture solution. The resulting slurry was filtered through a 300‐mesh nylon mesh, and the cells were then centrifuged at 350 g for 5 min.

### Muscle stem cell purification

**Figure 1:**
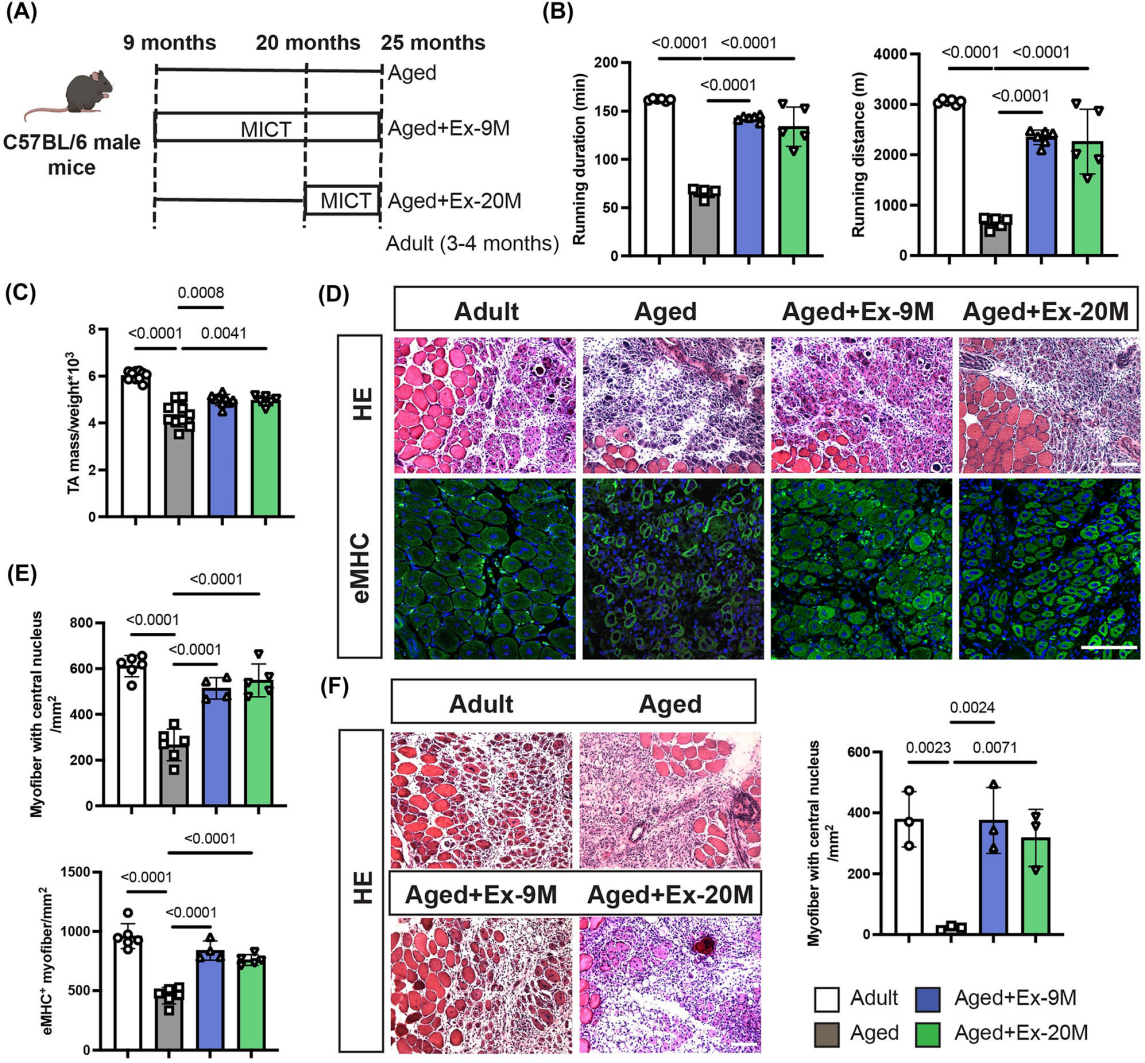
Aerobic exercise enhances skeletal muscle regeneration in aged mice. (A) Experimental setup illustration categorizing C57BL/6 male mice into groups: MICT initiated at 9 months old (aged + Ex‐9M), MICT initiated at 20 months old (aged + Ex‐20M) and controls for adult (3‐ to 4‐month‐old) and aged (25‐month‐old) mice. (B) Measurement of running duration and distance in the endurance test for 25‐month‐old and adult mice (*n* = 6 mice for the adult, aged and aged + Ex‐9M group; *n* = 5 for the aged + Ex‐20M group). (C) Tibialis anterior (TA) muscle weight was normalized to body weight (*n* = 10 mice for the adult, aged and aged + Ex‐9M group; *n* = 6 for the aged + Ex‐20M group). (D) Evaluation of muscle regeneration in TA muscles at 6 dpi. Representative haematoxylin and eosin (H&E) and embryonic myosin heavy chain (eMHC) stained TA tissue sections for adult, aged, aged + Ex‐9M and aged + Ex‐20M mice. Scale bar, 100 μm. (E) Quantification of the injured area occupied by central nuclear myofibers or eMHC^+^ myofibers in TA cross‐section (*n* = 6 mice for the adult and aged groups; *n* = 5 for the aged + Ex‐20M group; *n* = 4 for the aged + Ex‐9M group). (F) MuSCs isolated from adult, aged, aged + Ex‐9M and aged + Ex‐20M mice were transplanted into MuSC‐deleted mice. H&E staining of TA muscle 5 days after transplantation (6 dpi) and quantification results (*n* = 3 mice per group). Scale bar, 100 μm. Data are summarized with mean ± SD; one‐way ANOVA was used in panels (B), (C), (E) and (F).

MuSCs isolated from adult, aged, aged + Ex‐9M and aged + Ex‐20M groups included in *Figure* *1* were purified using a mixed approach involving a negative selection approach targeting CD31 (BD, #558738, 1:100), CD45 (BD, #553079, 1:100), Sca1 (BD, #557405, 1:100) and CD11b (BD, #557396, 1:100), and a positive selection targeting integrin (R&D Systems, #FAB3518A, 1:100) and CD34 (BD, #551387, 1:100), markers (magnetic‐activated cell sorting [MACS]). Initially, 1 μL each of CD31‐FITC, CD45‐FITC, SCA‐1‐FITC, CD34‐APC and integrin α7‐APC antibodies were added to 200 μL of the cell suspension. This mixture underwent a 30‐min incubation on ice or at 4°C. Subsequently, 1 mL of 2% foetal bovine serum (FBS) in Dulbecco's modified Eagle's medium (DMEM) was added to the cell suspension, followed by centrifugation at 350 g and 4°C for 3 min. This process was repeated twice to eliminate the supernatant. The resulting cell pellet was then suspended in 200 μL of 2% FBS in DMEM, and 10 μL of anti‐FITC beads (Miltenyi Biotec, #130‐090‐855) was added, followed by a 30‐min incubation at 4°C. Following this, 1 mL of MACS buffer was introduced, followed by additional rounds of centrifugation and washing. The pellet was suspended in 1.0 mL of MACS buffer and placed onto an LS column on a magnetic board. Flow‐through cells containing CD31‐FITC, CD45‐FITC and SCA‐1‐FITC‐positive cells were collected after centrifugation at 350 g and 4°C for 3 min. The collected cells were subjected to another round of incubation, this time with 10 μL of anti‐APC magnetic beads (Miltenyi Biotec, #130‐048‐701), followed by additional centrifugation and washing steps. For the final separation, the collected cells were loaded onto another LS column (Column No. 2) on the magnetic board, targeting integrin α7‐negative cells. Multiple washing steps were conducted using the MACS buffer. The collected cells were subsequently removed from the column, and after centrifugation at 500 g and 4°C for 3 min, the supernatant was discarded. The resultant cells were resuspended in 1 mL of culture medium and used for further analysis or sorting. The isolated MuSCs were then suspended in 2–4 mL of F‐10 primary myoblast growth medium.

For the other MuSCs mentioned in this article, a preplating technique was employed for purification. Following enzyme digestion, the cells were subjected to a preplating step on uncoated plates for 2 h. Subsequently, the supernatant containing the desired cells was transferred to a Matrigel‐coated plate for further experimentation. The purity of the cells was assessed using immunofluorescence (IF) staining.

### CCN2 overexpression and knockdown in muscle stem cells

MuSCs isolated from adult mice (3–4 months old, for CCN2 overexpression) or aged mice (25 months old, for CCN2 knockdown) were seeded into a six‐well plate at a density of 3 × 10^4^ cells per well and incubated overnight at 37°C. The following day, a mixture containing complete culture medium, transfection agent P (Gikai biological) and virus (Multiplicity of infection, MOI = 30) was prepared. The medium from the previous day was aspirated, and 1 mL of the prepared mixture was added to each well. After 18 h of transfection, the viral culture medium was replaced with fresh complete culture medium, and incubation continued at 37°C. At 72 h post‐transfection, the transfection efficiency was assessed using a fluorescence microscope. Subsequently, the viral culture medium was removed and replaced with fresh complete culture medium supplemented with 1 μg/mL puromycin for a 3‐day selection period. For CCN2 overexpression, we utilized lentivirus‐expressing exogenous mouse CCN2 (Lenti‐CCN2 group) along with the control virus (Lenti‐CON group). For CCN2 knockdown, we employed lentivirus‐expressing shRNA (caGGAAGATGTACGGAGACAT) targeted to CCN2 (Lenti‐CCN2shR) along with a negative control virus (Lenti‐NegshR).

### Muscle stem cell transplantation

At 3 months of age, *Pax7* ^CreERT2/+^; *Rosa26* ^DTA/+^ mice received daily intraperitoneal injections of tamoxifen at a dosage of 75 mg/kg of body weight in 100% corn oil for 7 days, followed by a subsequent waiting period of 7 days to deplete MuSCs. One day before receiving transplants, the TA muscles were injured with 10 μM of CTX (50 μL). Each transplant was performed in a volume of 30 μL over a single injection track using a Hamilton syringe (Gastight 1800 series). For the transplant of MuSCs isolated from adult, aged, aged + Ex‐9M and aged + Ex‐20M groups, 10 000 freshly isolated (passage 0) MuSCs were used. For the transplantation of Lenti‐Con and Lenti‐CCN2, Lenti‐Neg and Lenti‐CCN2shRNA MuSCs, 2 × 10^5^ cells were used for each mouse.

### Muscle stem cell culture

MuSCs were cultured with Ham's F‐10 medium (Sigma, #N6635) supplemented with 20% FBS (Sijiqing, #13011‐8611) and 100 U/mL penicillin and 100 μg/mL streptomycin (Sigma, #P4333‐100mL), along with 0.5% chicken embryo extract (US biological, #c3999) and 100 μg/mL basic fibroblast growth factor (bFGF) (Shanghai Yuanye, #S28686). The culture dish was coated with Matrigel (Corning, #354230).

### Histological analysis

Muscles were harvested from anaesthetized mice and immediately fresh‐frozen in liquid nitrogen‐cooled isopentane. Transverse 7‐μm sections were produced and fixed with 4% paraformaldehyde. These sections were then subjected to staining with haematoxylin and eosin (H&E), Masson's trichrome stain or specific antibodies. For immunostaining, cell samples were directly fixed with 4% paraformaldehyde. With or without heat‐induced epitope retrieval (HIER) (10 mM of sodium citrate, 0.05% Tween‐20, pH 6.0, 95°C for 10 min), the samples were incubated with PBS containing 0.25% Triton X‐100 for 20 min, three times. Afterwards, the samples were incubated with 5% bovine serum albumin (BSA) and Fab fragment antibodies for 1 h to block the unspecific binding of the antibodies. Subsequently, the samples were stained overnight for Pax7 (DSHB, #AB_528428, 1:50), Ki‐67 (Abcam, #ab15580, 1:100), BrdU (Abcam, #ab6326), embryonic myosin heavy chain (eMHC) (DSHB, #F1.652, 1:50) or myosin heavy chain (MHC) (Santa Cruz, #sc‐32732, 1:100). DAPI was used to visualize nuclei. Sections were imaged with a Zeiss 800 confocal microscope. Pax7‐positive cells and eMHC‐positive fibre numbers were quantified manually using ImageJ.

### Western blots

Muscle homogenates were centrifuged, and the protein content of the supernatant was quantified using the BCA Protein Assay Kit (Beyotime Biotechnology). Equal amounts of protein (20 μg) were separated on 8–10% sodium dodecyl sulfate (SDS)–polyacrylamide gel electrophoresis gels and transferred to polyvinylidene difluoride (PVDF) membranes. The membranes were incubated overnight at 4°C with primary antibodies recognizing collagen I (CST, #72026s), CCN2 (Santa Cruz, #SC‐365970, 1:100), aSMA (Santa Cruz, #sc‐53142), MyoD (Santa Cruz, #SC‐377460, 1:100), Myomixer (R&D Systems, #AF4580), MHC (Santa Cruz, #SC‐32732, 1:200), tubulin (Santa Cruz, #SC‐58666, 1:500) and GAPDH (Santa Cruz, #SC‐365062‐hrp, 1:500). After three washes with PBS with Tween‐20 (PBST), the membranes were incubated with secondary antibodies for 60 min at room temperature. Signals were detected using a ChemiDoc Touch (Bio‐Rad) using enhanced chemiluminescence (ECL) detection reagents (CWBIO).

### Reverse transcription quantitative PCR (RT–qPCR)

RNA was isolated using the RNAiso Plus (Takara, #9109) and reverse transcribed using the PrimeScript™ RT reagent kit with the gDNA Eraser (Takara, #RR047A). qPCR was performed with the qPCR detection system (Bio‐Rad) by using TB green Premix Ex Taq™ (Takara, #RR420A). Technical triplicate Ct values were averaged and normalized to the mean of GAPDH and 18s housekeeping transcript values. Within each experiment, expression levels were normalized to the mean control level. Primer sequences used were as follows (from 5′ to 3′): col1a1 forward, GCAAGAGGCGAGAGAGGTTT; col1a1 reverse, GACCACGGGCACCATCTTTA; GAPDH forward, TGGAAAGCTGTGGCGTGATG; GAPDH reverse, GTCAGATCCACGACGGACAC; TCF4 forward, GATGGATGAACCCGGCAAAC; TCF4 reverse, CACTGCTTACAGGAGGCGAA; CCN2 forward, AGAACTGTGTACGGAGCGTG; CCN2 reverse, GTGCACCATCTTTGGCAGTG.

### RNA sequencing

The purified MuSCs were cultured in vitro for three passages to obtain a sufficient number of cells, and the third passage was utilized for RNA‐seq. The RNA‐seq process was carried out by BGI Genomics Co. using the BGISEQ platform with a PF150 sequencing length. Clean reads were aligned to the reference genome and genes using HISAT and Bowtie 2, respectively. The analysis of differentially expressed genes (DEGs) was conducted for genes with a normalized FPKM (fragments per kilobase per million mapped fragments) with a false discovery rate (FDR) of ≤0.05. Subsequently, the DEGs underwent further analysis using Gene Set Enrichment Analysis (GSEA).

### Digital image acquisition and processing

Digital images were acquired using different imaging systems: (1) an inverted microscope equipped with a camera for capturing immunohistochemical colour pictures and (2) a Zeiss 800 confocal microscope for capturing IF pictures. To quantify central nuclear fibres (CNFs), fibrosis and TCF4‐positive cell numbers, four to five images covering the injured area were captured for each mouse, and the average of these data was used for analysis. For counting eMHC‐positive fibres, three to four images of the central area of injury were captured. Cross‐sectional area (CSA) and Pax7‐positive cell numbers were calculated from four randomly captured images per mouse, and their averages were used for analysis. Images for other experiments were captured randomly. Furthermore, the person responsible for imaging all mouse samples was blinded to the sample group identities. However, for other experiments, the investigators were not blinded.

### Statistical analysis

The sample size (*n*) for each experimental group is provided in the figure legend. GraphPad Prism software was employed for statistical analyses. Quantitative data, presented as histograms, are expressed as means ± standard deviations (SDs), with error bars representing the variability. The results from each group were averaged for descriptive statistics. For comparisons between two groups, Student's *t*‐test was utilized; for multiple comparisons, a one‐way analysis of variance (ANOVA) was performed, with statistical significance set at *P* < 0.05.

## Results

### Aerobic exercise enhances skeletal muscle regeneration in aged mice

To explore the impact of long‐term aerobic exercise on MuSC function and muscle regeneration, adult mice were categorized into groups undergoing MICT starting at 9 months old (aged + Ex‐9M) or 20 months old (aged + Ex‐20M). An age‐matched control group (aged) and an adult group (3–4 months old) were also included (*Figure* *1* *A*). The maximum speed of mice, coupled with MICT, exhibited a gradual increase from 10 months old (33.7 ± 4.19 m/min) to 14 months old (40.5 ± 2.55 m/min), followed by a decline up to 19 months old (32.2 ± 1.93 m/min). Subsequently, there was a slight upregulation, succeeded by another decline (*Figure* *S1* *a*). Notably, mice starting MICT at 9 months old showed a significantly higher maximum speed during the period of 21–23 months old compared with those initiating MICT at 20 months old (*Figure* *S1* *a*). By 25 months of age, the running duration (161.9 ± 1.29 and 65.33 ± 4.32 min in adult and aged mice, respectively) in the endurance performance test of aged mice had decreased to 2/5 of adult mice, and the running distance changed from 3058.28 ± 46.26 m in adult mice to 659.17 ± 103.64 m in aged mice (*Figure* *1* *B*). However, MICT mitigated the declining trend in maximum running duration and distance (*Figure* *1* *B*). Furthermore, aging was associated with a decrease in the mass/body weight of the TA (*Figure* *1* *C*) and quadriceps femoris muscles (*Figure* *S1* *b*), as well as a reduction in muscle fibre CSA (*Figure* *S1* *c*). MICT, on the other hand, led to an increase in these parameters compared with the aged group (*Figures* *1* *C* and *S1* *b,c*). Although MICT did not alleviate the decline in the number of MuSCs, as evidenced by the cell positivity for Pax7 (a specific marker for MuSCs) in aged mice (*Figure* *S1* *d*), it did contribute to a reduction in DNA damage in gammaH2AX‐labelled MuSCs (*Figure* *S1* *e*).

To evaluate the impact of exercise on the regenerative potential of MuSCs, TA muscles underwent CTX‐induced injury after 16 or 5 months of exercise intervention. Following a 5‐day recovery period (6 days post‐injury [6 dpi]), histological examination was conducted on TA muscles. A comparison with adult mice revealed that old mice had fewer newly formed muscle fibres, characterized by CNFs or myofibers expressing eMHC, a protein found in foetal and regenerating muscle fibres (*Figure* *1* *D,E*). In the adult group, an average of 610.82 ± 46.76 CNFs/mm^2^ was observed, whereas in the older group, this number dropped to approximately 266.35 ± 68.66/mm^2^ (*Figure* *1* *D,E*). However, implementation of MICT increased the number of CNFs to 513.42 ± 47.1 and 548.29 ± 71.82/mm^2^ in aged + Ex‐9M and aged + Ex‐20M groups, respectively (*Figure* *1* *D,E*). MICT also increased the number of eMHC‐positive myofibers in aged mice (*Figure* *1* *D,E*). These data demonstrated that exercise significantly expedited the regeneration capacity of old mice, bringing it closer to that of their younger counterparts (*Figure* *1* *D,E*), indicating that exercise may mitigate the aging process in MuSCs, given their primary role in skeletal muscle regeneration.

To explore the extent to which exercise rejuvenates muscle repair through intrinsic changes in MuSCs, we transplanted MuSCs isolated from adult, old or exercised mice to *Pax7* ^CreERT2/+^; *Rosa26* ^DTA/+^ mice. These recipient mice had MuSCs depleted through intraperitoneal injection with tamoxifen. Pax7 IF staining was utilized to label the MuSCs and assess the deletion efficiency. Without tamoxifen injection, the MuSC count per field was 3.76, whereas post‐tamoxifen injection, it dropped significantly to 0.16, demonstrating a depletion efficiency of 95.7% (*Figure* *S2a*). Furthermore, following CTX injury, which leads to a deficiency in regeneration, MuSCs were absent in tamoxifen‐injected mice but present in mice without tamoxifen injection (*Figure* *S2b*). A day before transplant, CTX injury was performed on the TA of MuSC‐depleted mice. After 5 days post‐transplant, TA muscles were isolated and sectioned for staining. In comparison to adult donor MuSCs, aged donor MuSCs resulted in the formation of fewer and smaller myofibers. Exercise for 9 or 20 months significantly restored these parameters (*Figure* *1* *F*) and increased the cell proliferation rate of MuSCs (*Figure* *S1* *f*). Collectively, these experiments demonstrate that MICT enhances the functionality of aged MuSCs.

### Elevated CCN2 expression in aged muscle stem cells impairs muscle regeneration ability

**Figure 2:**
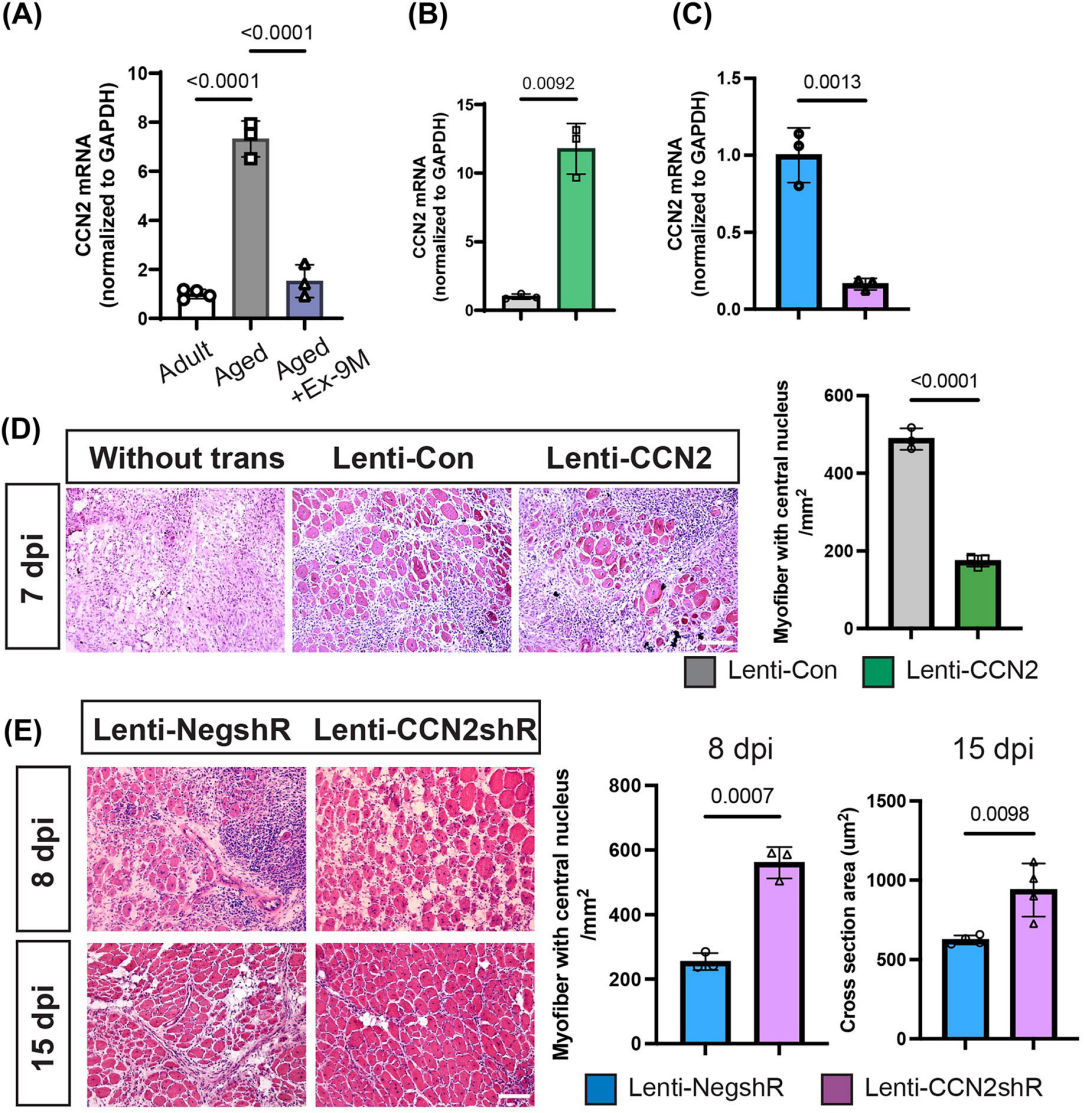
Elevated CCN2 expression in aged MuSCs impairs muscle regeneration ability. (A) RT–PCR results depicting the expression levels of CCN2 in MuSCs of adult, aged and aged + Ex‐9M groups (*n* = 3 per group). (B, D) MuSCs isolated from adult mice were transfected with a control (Lenti‐CON) or CCN2‐overexpressed (Lenti‐CCN2) lentivirus. (B) RT–PCR result of CCN2 mRNA expression. (C, E) MuSCs isolated from 25‐month‐old mice were subjected to lentiviral transfection with virus expressing shRNA targeted to CCN2 (Lenti‐CCN2shR) or the control virus (Lenti‐NegshR). (C) RT–PCR result of CCN2 mRNA expression level in Lenti‐CCN2shR and Lenti‐NegshR groups. (D) Illustration of H&E staining of TA cross‐sections transplanted with control or CCN2‐overexpressed MuSCs at 7 dpi, along with the corresponding statistical results (*n* = 3 per group). Scale bar, 100 μm. (E) H&E staining of TA cross‐sections transplanted with Lenti‐NegshR or Lenti‐CCN2shR MuSCs at 8 and 15 dpi and statistical results (*n* = 3 for 8 dpi; *n* = 4 for 15 dpi). Scale bar, 100 μm. Data are summarized with mean ± SD; one‐way ANOVA was used in panel (A); and a *t*‐test was used in panels (B)–(E).

To determine the global differences between the transcriptomes of adult and aged MuSCs, we conducted RNA‐seq for MuSCs isolated from adult and aged mice. GSEA revealed that MuSCs from aged mice exhibited heightened activation of genes associated with ECM–receptor interaction and focal adhesion compared with adult mice (*Figure* *S3a,b*). Analysis of the potential upstream regulators of these DEGs identified connective tissue growth factor (CTGF)/CCN2 as a key upstream gene. Consistent with previous studies showing significant alterations in the expression of genes related to the transforming growth factor‐beta (TGF‐β) signalling pathway in aged MuSCs, including a substantial upregulation of CCN2,^19^ our sequencing data revealed increased CCN2 expression in aged mice compared with adult mice. RT–qPCR further confirmed the upregulation of CCN2 in aged MuSCs, and its expression was downregulated by MICT (*Figure* *2* *A*). To further elucidate the role of CCN2 in the regeneration process, we performed CCN2 overexpression and knockdown experiments. Specifically, we induced CCN2 overexpression in adult MuSCs (Lenti‐CCN2, Lenti‐CON as a control) and knockdown in aged MuSCs (Lenti‐CCN2shR, Lenti‐NegshR as a control) isolated from 25‐month‐old mice using lentiviral transfection. CCN2 overexpression significantly increased CCN2 expression (*Figure* *2* *B*), while knockdown reduced it (*Figure* *2* *C*). Subsequently, these MuSCs were transplanted into MuSC‐depleted mice. H&E staining showed that there were approximately 488.07 ± 27.63 CNFs/mm^2^ in the Lenti‐CON group and only ~173.99 ± 14.28 CNFs/mm^2^ in the Lenti‐CCN2 group at 7 dpi (*Figure* *2* *D*). For CCN2 knockdown, at 8 dpi, H&E staining revealed approximately 254.5 ± 26.36/mm^2^ CNFs in the Lenti‐NegshR group, whereas CCN2 knockdown significantly increased this number to 560.39 ± 48.71/mm^2^ (*Figure* *2* *E*). By 15 dpi, the CSA of the Lenti‐CCN2shRNA group exceeded that of the Lenti‐NegshR group (*Figure* *2* *E*). These data demonstrate that CCN2 overexpression in adult MuSCs impairs myofiber formation, while knockdown of CCN2 in aged MuSCs rescues its impaired regeneration capacity.

### CCN2 inhibits the late differentiation of muscle stem cells

**Figure 3:**
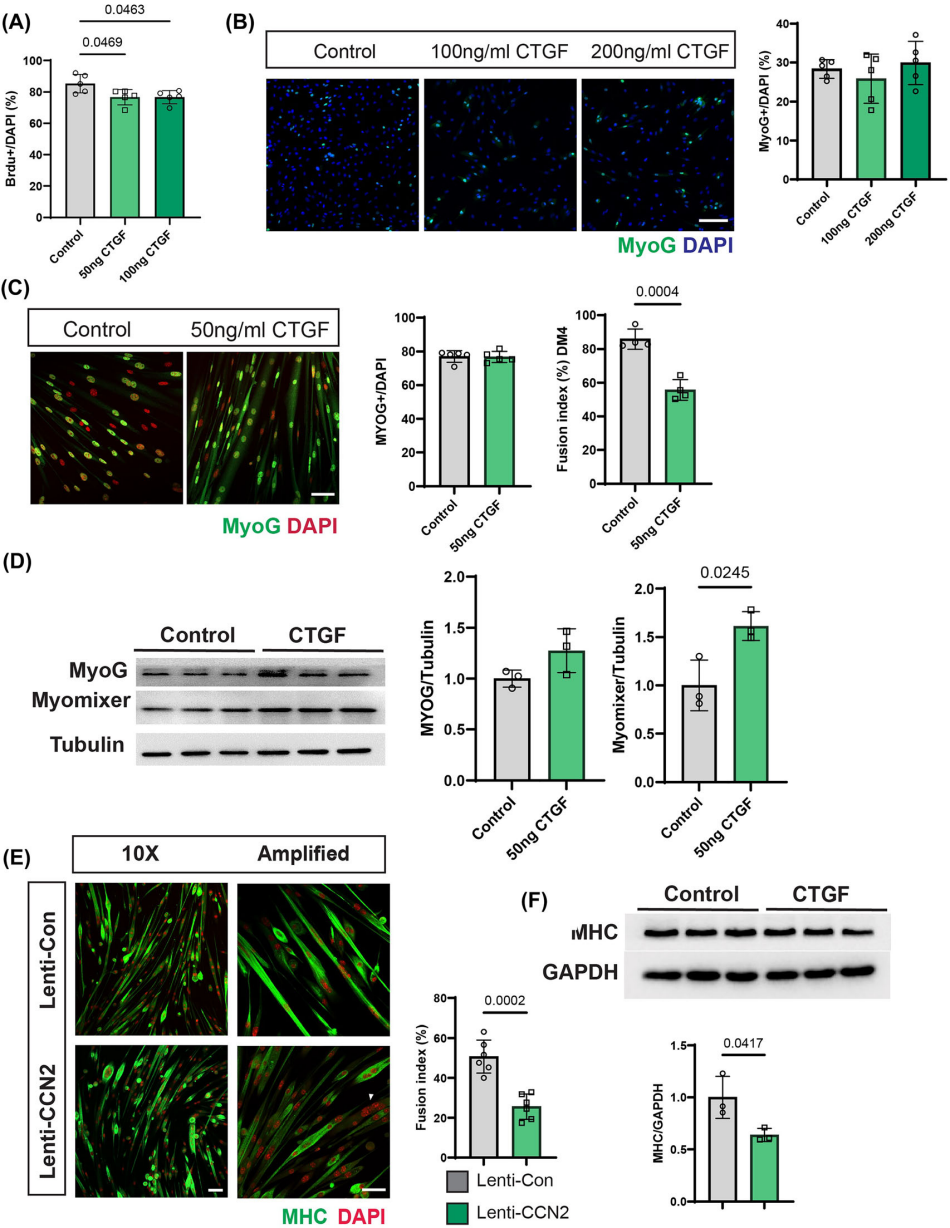
Inhibition of MuSC late differentiation by administration of recombinant CCN2/CTGF protein or CCN2 overexpression. (A–D) MuSCs were isolated from adult mice and treated with different concentrations of recombinant CTGF protein or control in vitro. (A) BrdU incorporation in the control and CTGF protein supplement groups. Recombinant CTGF protein was administered to MuSCs isolated from adult mice for 2 days, with BrdU added 24 h before sample harvest. (B) Immunofluorescence staining of MyoG representing autonomously differentiated MuSCs in the control and CTGF‐treated groups. MuSCs were cultured with growth medium with/without CTGF supplement and quantification results. Scale bar, 100 μm. (C) IF staining of MyoG after 4 days of differentiation and quantification results, including the fusion index after 4 days of differentiation (*n* = 5 per group in panels B and C). Scale bar, 50 μm. (D) MyoG and Myomixer western blot results. MyoG and Myomixer protein expression levels were normalized to tubulin (*n* = 3 per group). (E, F) MuSCs from adult mice were transfected with a control (Lenti‐CON) or CCN2‐overexpressed (Lenti‐CCN2) lentivirus. (E) Four days after differentiation, cells were stained with MHC and DAPI, and the fusion index was quantified as myotubes with three or more nuclei/total nuclei number (*n* = 6 per group). Scale bar, 50 μm. (F) Western blot results of MHC normalized to GAPDH (*n* = 3 per group). Data are presented as mean ± SD; one‐way ANOVA was used in panels (A) and (B); and a *t*‐test was used in panels (C)–(F).

To investigate the effect of CCN2 on MuSCs, we treated MuSCs with recombinant CCN2/CTGF protein in cell culture. Compared with the control group, the administration of recombinant CTGF protein slightly inhibited MuSC proliferation, as indicated by reduced BrdU incorporation (*Figure* *3* *A*). However, it did not affect autonomous cell differentiation, as demonstrated by comparable levels of MyoG expression between the treated and control groups (*Figure* *3* *B*). Interestingly, administration of recombinant CTGF protein inhibited cell fusion, as evidenced by a reduced fusion index from 85.84 ± 5.98 to 55.74 ± 6.18, without impacting early differentiation, as shown by the percentage of MyoG‐positive cells (*Figure* *3* *C*). Western blot analysis revealed no significant difference in MyoG protein expression levels between the treated and control groups (*Figure* *3* *D*), but CCN2 protein administration led to a slight increase in Myomixer expression (*Figure* *3* *D*). Similarly, CCN2 overexpression in MuSCs resulted in inhibited fusion (*Figure* *3* *E*). Furthermore, we observed myotubes without MHC expression in CCN2‐overexpressing MuSCs (*Figure* *3* *E*), indicating suppression of late‐stage differentiation. Consistently, MHC protein expression was inhibited by CCN2 overexpression (*Figure* *3* *F*), confirming that CCN2 suppresses the late differentiation of MuSCs, as MHC serves as a marker for late‐stage differentiation. Overall, these findings demonstrate that CCN2 inhibits the late differentiation of MuSCs.

### CCN2 induces the transition of muscle stem cells from a myogenic to a fibrogenic state

**Figure 4:**
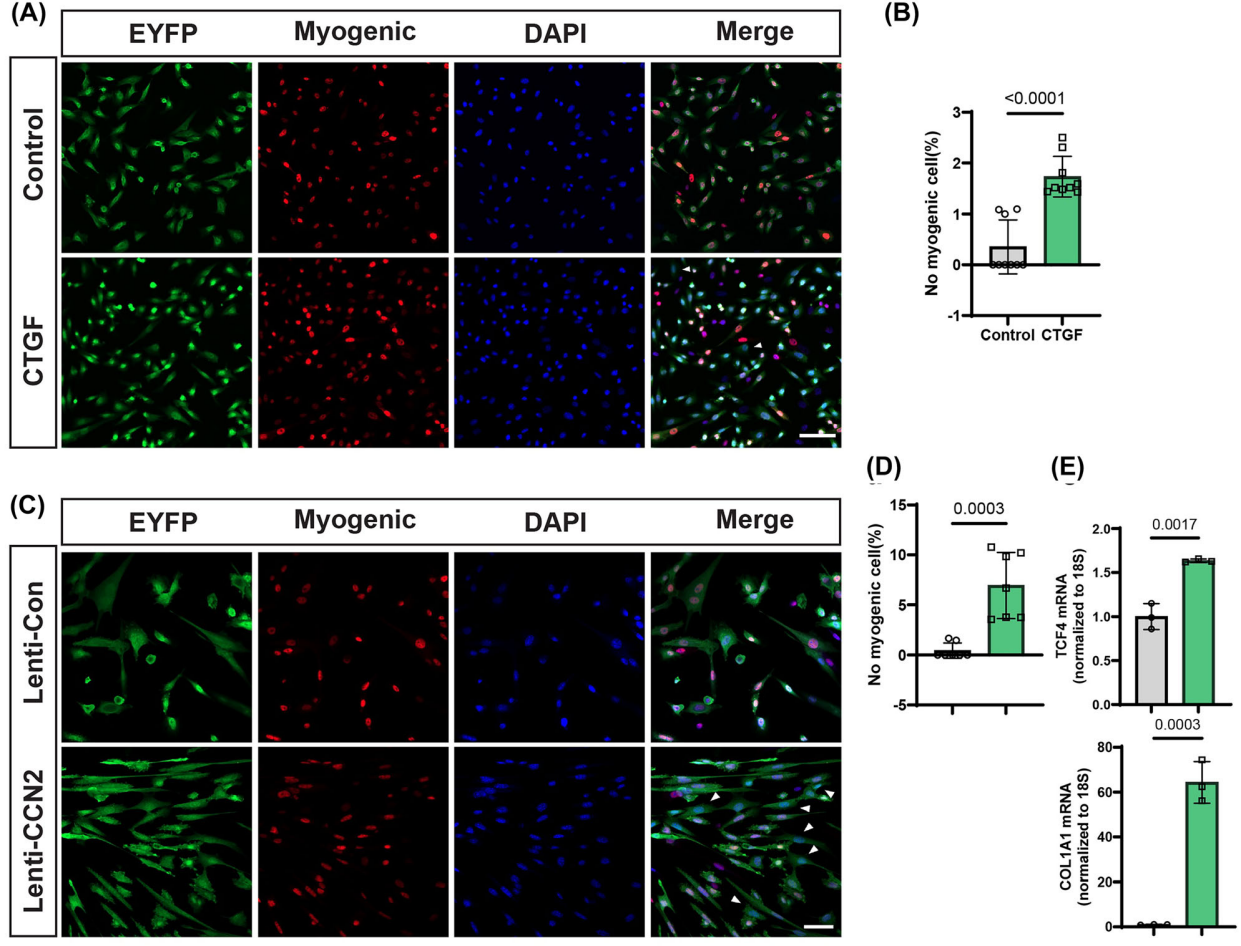
CCN2 induces increased myogenic‐to‐fibrous conversion of MuSCs. (A) MuSCs isolated from adult *Pax7* ^CreERT2/+^;*R26R* ^EYFP/+^mice were cultured with/without 100 ng/mL CCN2/CTGF for two passages and then stained for myogenic markers Pax7, MyoD, MyoG and YFP to identify nonmyogenic YFP‐positive cells (white arrowheads). Scale bar, 100 μm. (B) Quantification of the percentage of YFP‐positive cells that were nonmyogenic (*n* = 7 per group). (C) MuSCs from adult *Pax7* ^Cre^ ^ERT2/+^;*R26R* ^EYFP/+^ mice were transfected with control (Lenti‐CON) or CCN2‐overexpressed (Lenti‐CCN2) lentivirus, stained for myogenic markers, and YFP to identify nonmyogenic cells (white arrowheads). Scale bar, 50 μm. (D) Quantification of the percentage of YFP‐positive cells that were nonmyogenic (*n* = 7 per group). (E) RT–qPCR for fibroblast markers TCF4 and collagen I in MuSCs in Lenti‐CON and Lenti‐CCN2 groups (*n* = 3 per group). Data are summarized with mean ± SD; a *t*‐test was used in panels (B), (D) and (E).

Previous studies have revealed that in aged and DMD (Duchenne muscular dystrophy) mice, MuSCs tend to undergo a transition from a myogenic to a fibrogenic state, serving as a source of fibroblasts and impairing the regeneration ability of MuSCs.^13^, ^15^ A previous study has shown that CCN2 induces C2C12 cell dedifferentiation, characterized by downregulating MyoD and desmin, two markers of committed myoblasts.^20^ To investigate whether CCN2 induces MuSCs to transition from a myogenic to a fibrogenic state, the *Pax7* ^CreERT2/+^;*R26R* ^EYFP/+^ mouse strain was utilized. In this strain, the yellow fluorescent protein (YFP) gene is expressed in satellite cell progeny cells following tamoxifen administration, enabling genetic lineage tracing. Incubating MuSCs isolated from *Pax7* ^CreERT2/+^; *R* *26R* ^E^ ^YFP/+^ mice with mouse recombinant CCN2 protein resulted in 1.6% of YFP‐positive cells that did not express any myogenic proteins, including Pax7, MyoD and myogenin (*Figure* *4* *A,B*). Overexpression of CCN2 by lentivirus resulted in 7% of YFP‐positive cells that did not express any myogenic proteins (*Figure* *4* *C,D*). Concurrently, the transcription level of the fibroblast marker gene TCF4 increased (*Figure* *4* *E*). mRNA levels of type I collagen were 60 times higher than those in cells transfected with a control virus (*Figure* *4* *E*). This suggests that CCN2 can promote the conversion of MuSCs from a myogenic to a fibrogenic state (*Figure* *4* *A–E*).

### Muscle stem cell‐secreted CCN2 induces fibroblast overproliferation and skeletal muscle fibrosis

**Figure 5:**
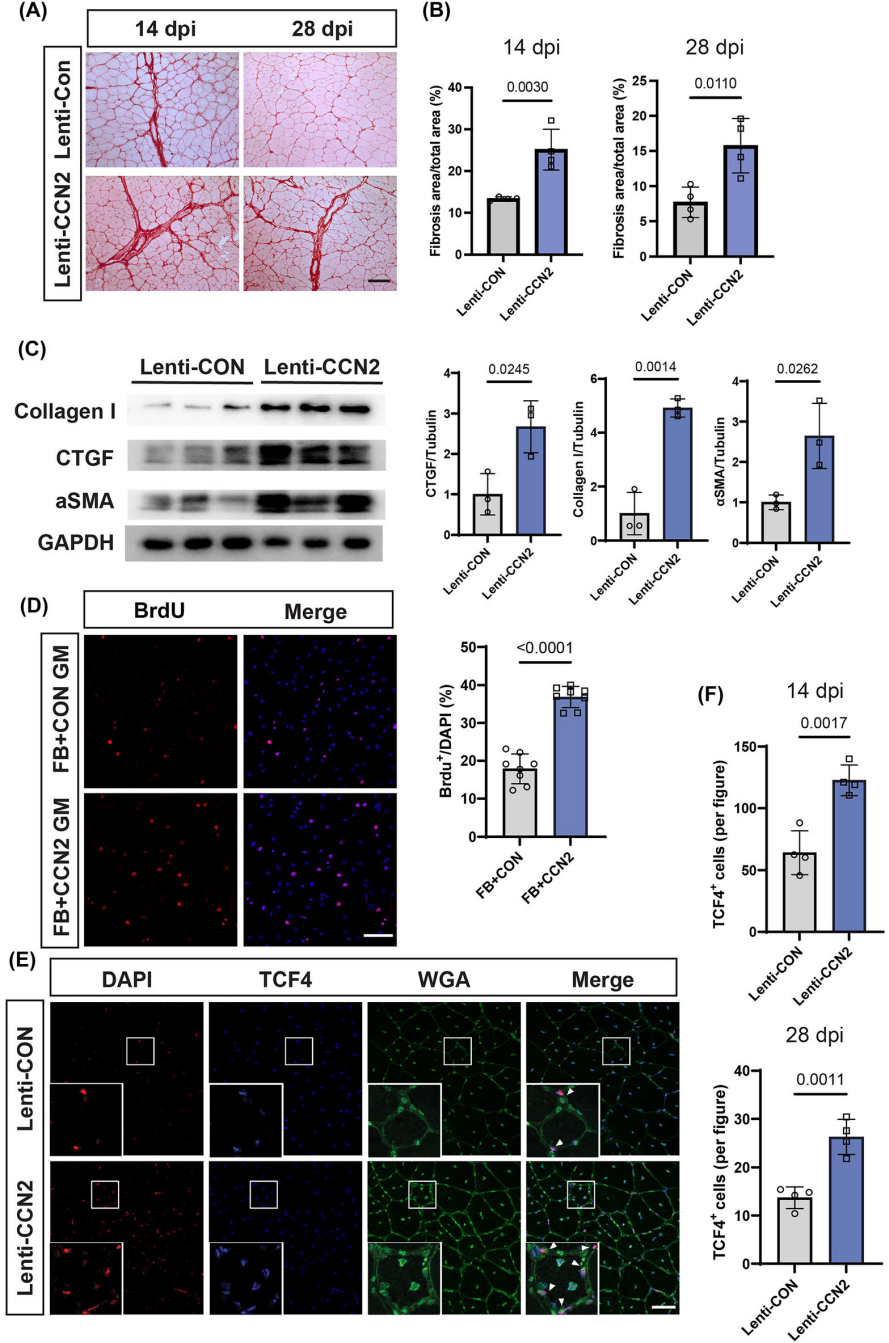
MuSC‐secreted CCN2 leads to fibroblast overproliferation and skeletal muscle fibrosis. (A‐C, E, F) TA muscles transplanted with control MuSCs (Lenti‐CON) or CCN2‐overexpressed MuSCs (Lenti‐CCN2). (A) At 14 and 28 dpi, the TA muscle cross‐section was stained with Sirius Red. Scale bar, 100 μm. (B) Quantification of collagen proportion after Sirius Red staining (*n* = 4 per group). (C) Western blot analysis of collagen I, aSMA and CCN2 in TA at 14 dpi and quantification results (*n* = 3 mice per group). (D) BrdU staining in fibroblast cultures with growth medium derived from control MuSCs (FB + CON GM) or CCN2‐overexpressed MuSCs (FB + CCN2 GM), and the results were quantified (*n* = 8 per group). Scale bar, 100 μm. (E) Cross‐sections of TA muscles at 14 dpi were stained for TCF4, WGA and DAPI in adult and aged mice. Scale bar, 50 μm. (F) TCF4‐positive cell numbers were counted at 14 and 28 dpi (*n* = 4 per group). Data are summarized with mean ± SD; a *t*‐test was used in panels (B)–(D) and (D).

MuSCs derived from aged mice undergo a phenotypic shift from myogenic to fibrogenic lineage, impairing muscle regeneration and promoting a fibrotic response.^13^ To examine whether elevated CCN2 expression in MuSCs contributes to skeletal muscle fibrosis, we transplanted Lenti‐CON and Lenti‐CCN2 MuSCs into preinjured TA muscles. TA muscle sections stained with Sirius Red at 14 and 28 days post‐barium chloride (BaCl_2_) injury revealed excessive collagen deposition and skeletal muscle fibrosis in mice receiving Lenti‐CCN2 MuSCs (*Figure* *5* *A,B*). Western blot analysis further confirmed elevated protein expression of collagen I, alpha‐SMA and CCN2 in the Lenti‐CCN2‐transplanted group at 14 dpi (*Figure* *5* *C*). Fibrosis results from the excessive proliferation of fibroblasts, which subsequently differentiate into myofibroblasts responsible for collagen production.^21^ Given that CCN2 is known as a profibrotic factor that stimulates fibroblast proliferation, we investigated whether CCN2 secreted from MuSCs enhances fibroblast proliferation. Initially, we utilized conditional medium from CCN2‐overexpressing MuSCs or control cells to culture primary fibroblasts isolated from young mouse skeletal muscle. Fibroblast culture with control MuSC medium showed 17% BrdU‐positive cells, whereas the BrdU‐positive cells cultured in Lenti‐CCN2 MuSC conditional medium reached 35%, twice as much as the control (*Figure* *5* *D*). This suggests that the elevated CCN2 expression in MuSCs leads to the rapid proliferation of fibroblasts. To further examine the effects of MuSCs overexpressing CCN2 on fibroblast proliferation in vivo, the TCF4‐positive fibroblast number was quantified at 14 or 28 dpi. And the data revealed that the number of TCF4‐positive fibroblasts in the Lenti‐CCN2 group remained twice as much as the Lenti‐Con group (*Figure* *5* *E,F*), indicating that CCN2 overexpression in MuSCs promotes fibroblast proliferation both in vitro and in vivo. Excessive proliferation and differentiation of fibroblasts result in increased collagen production and accumulation, leading to skeletal muscle fibrosis.

### Knockdown of CCN2 in aged muscle stem cells inhibits skeletal muscle fibrosis

**Figure 6:**
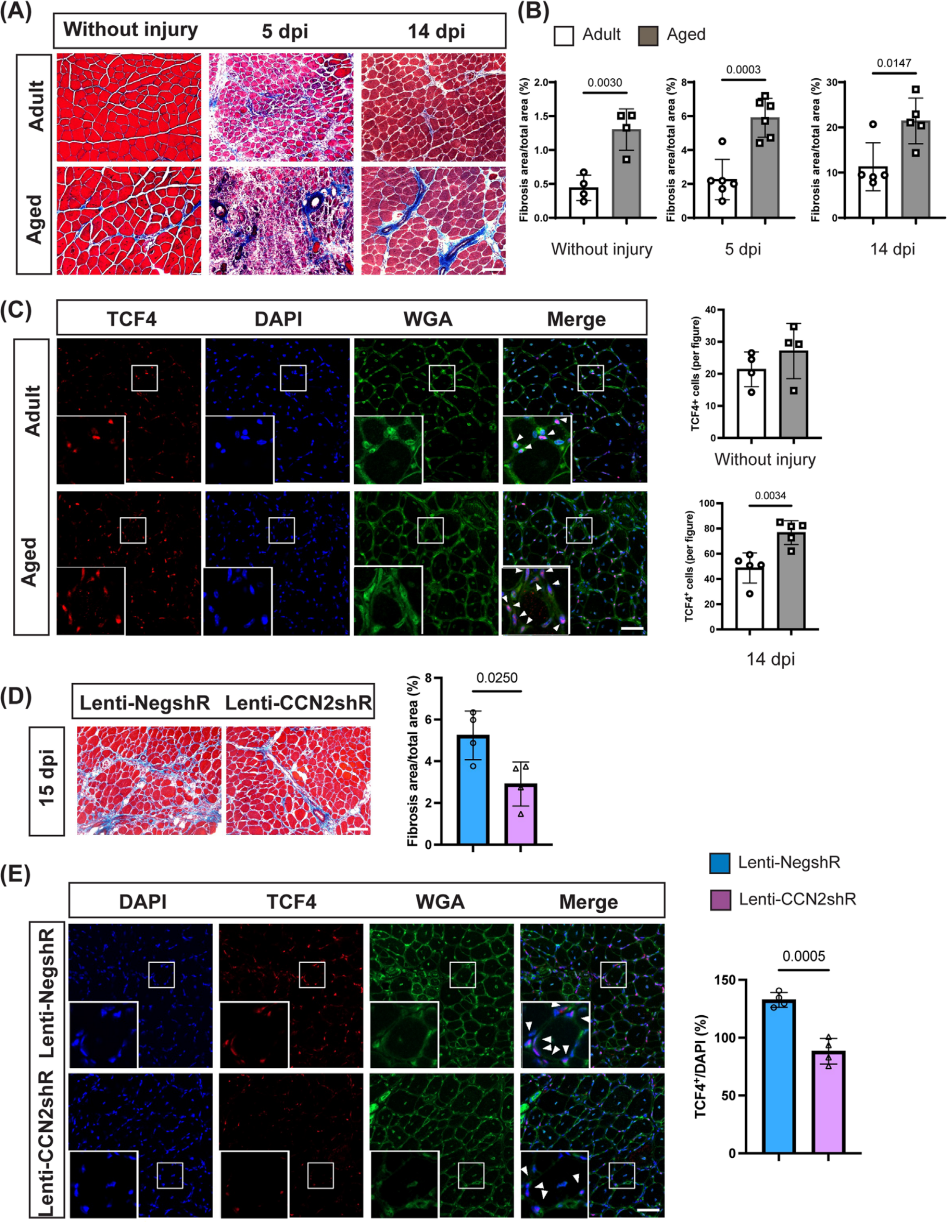
Knockdown of CCN2 in aged MuSCs inhibits skeletal muscle fibrosis. (A) Masson staining performed on TA muscles of adult (3–4 months old) and aged mice (25 months old) before, 5 and 14 dpi. Scale bar, 100 μm. (B) Quantification of fibrosis area from (A), *n* = 4 per group for without injury, *n* = 6 per group for 5 dpi and *n* = 5 per group for 14 dpi. (C) TCF4 staining to label fibroblasts, WGA for membrane, and DAPI for cell nuclei in TA muscle of adult and aged mice 14 dpi and quantification of TCF4‐positive cell numbers before and 14 dpi, *n* = 4 per group for without injury and *n* = 5 per group for 14 dpi. Scale bar, 50 μm. (D, E) TA muscle transplanted with Lenti‐NegshR and Lenti‐CCN2shR MuSCs was assessed at 15 dpi. (D) Masson staining of TA cross‐sections and quantification result, *n* = 4 per group. Scale bar, 100 μm. (E) TCF4, WGA, DAPI co‐staining of TA muscle and TCF4‐positive cell percentage, *n* = 4 per group. Scale bar, 50 μm. Data are summarized with mean ± SD; a *t*‐test was used in panels (B)–(E).

The upregulated expression of CCN2 in aged MuSCs contributes to an increased myogenic‐to‐fibrous conversion of MuSCs. Moreover, CCN2 secreted by MuSCs stimulates fibroblast proliferation. To assess the impact of these processes on skeletal muscle fibrosis, we examined fibrotic responses in aged mice before and after injury. Aged mice exhibited elevated fibrosis levels even without injury, as depicted in *Figure* *6* *A,B*, in comparison to their adult counterparts. This heightened fibrotic response was particularly evident at 5 and 14 dpi induced by BaCl_2_ injection in aged mice (*Figure* *6* *A,B*). Under uninjured conditions, there was no significant difference in the number of TCF4‐positive fibroblasts between adult and aged mice (*Figure* *6* *C*). However, following injury, the number of TCF4‐positive cells in aged mice significantly exceeded that in adult controls (*Figure* *6* *C*). To evaluate the effect of downregulating CCN2 expression in aged MuSCs on skeletal muscle fibrosis, we transplanted Lenti‐NegshR and Lenti‐CCN2shR MuSCs into MuSC‐depleted mice. At 15 dpi, Masson staining revealed a reduced fibrosis area in mice receiving Lenti‐CCN2shR MuSCs compared with those receiving Lenti‐NegshR MuSCs (*Figure* *6* *D*). Additionally, TCF4 IF staining demonstrated a decreased count of fibroblast cells in the Lenti‐CCN2shR group (*Figure* *6* *E*). These findings underscore the potential of suppressing CCN2 expression in aged MuSCs to enhance their regeneration capacity and alleviate skeletal muscle fibrosis post‐injury. Taken together, our results suggest that the elevated expression of CCN2 in aged MuSCs may promote skeletal muscle fibrosis following injury through two mechanisms: first, by inducing the conversion of MuSCs from a myogenic to a fibrogenic lineage and, second, by stimulating fibroblast proliferation.

### AICAR has the potential to replicate the effects of aerobic exercise on fibrosis following skeletal muscle injury in aged mice

**Figure 7:**
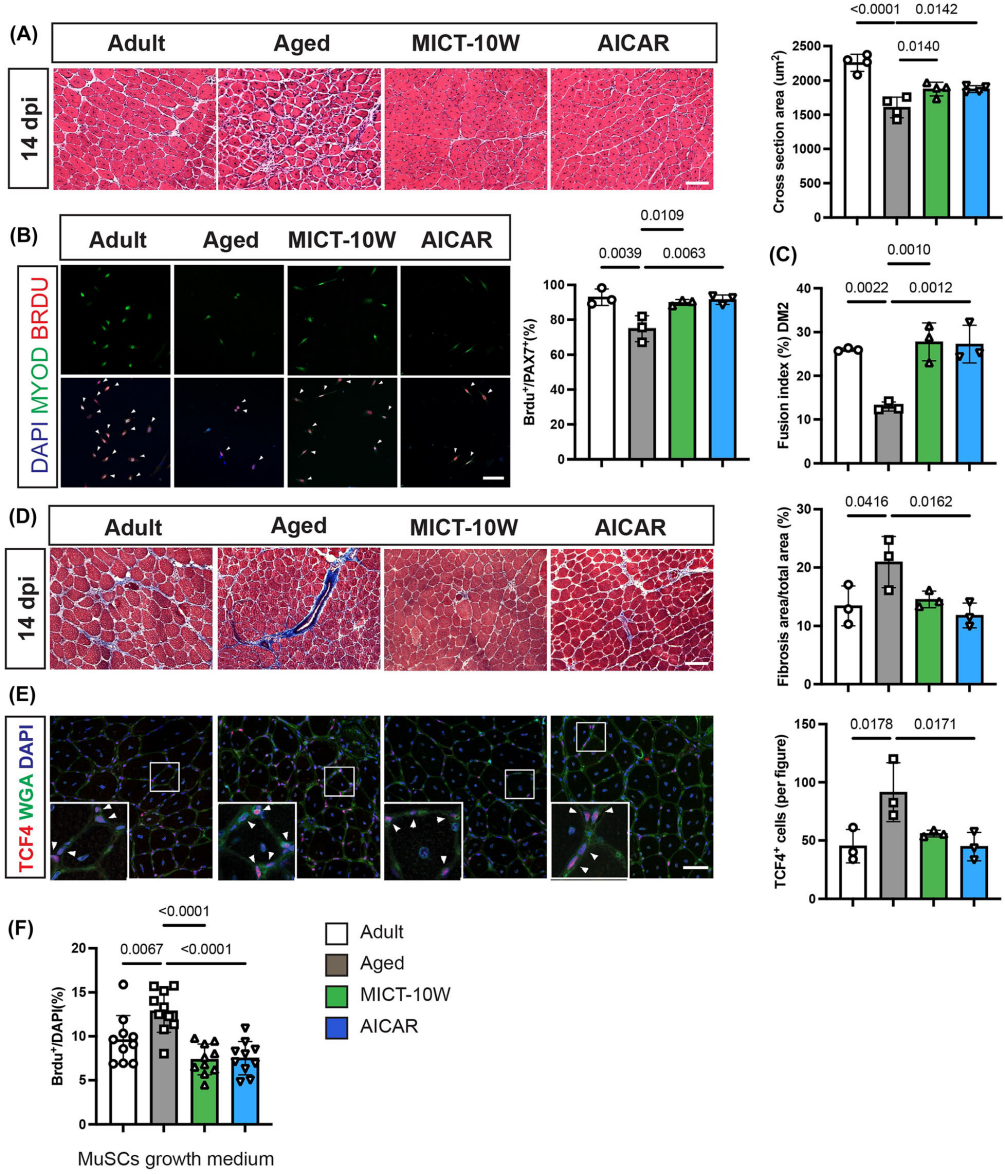
AICAR mimics the effects of aerobic exercise on skeletal muscle regeneration injuries in aged mice. Twenty‐three‐month‐old mice were subjected to MICT training (MICT‐10W) or AICAR administration for 10 weeks. (A) H&E staining of TA cross‐sections at 14 dpi and quantification of myofiber cross‐sectional area (CSA) (*n* = 4 per group). Scale bar, 100 μm. (B) In vitro culture of MuSCs isolated from adult, aged, MICT‐10W and AICAR groups. BrdU incorporation assessed activation ability and quantification results of BrdU and Pax7 double‐positive cells to Pax7‐positive cells (*n* = 3 per group). Scale bar, 100 μm. (C) Fusion index of 2 days after differentiation (*n* = 3 per group). (D) Masson staining of TA cross‐sections at 14 dpi and quantification results of fibrosis areas (*n* = 3 per group). Scale bar, 100 μm. (E) TCF4, WGA, DAPI co‐staining of TA muscle 14 dpi and TCF4‐positive cell number per figure (*n* = 3 per group). Scale bar, 50 μm. (F) Primary fibroblasts isolated from adult mice were cultured with medium derived from adult, aged, AICAR and MICT‐10W groups, demonstrating fibroblast cell proliferation by BrdU‐positive cell number to total cell number. Data are summarized with mean ± SD; one‐way ANOVA was used in panels (A)–(F).

Given that exercise is a well‐established activator of AMP‐activated protein kinase (AMPK),^22^ we utilized the AMPK agonist AICAR to investigate whether AMPK activation can improve skeletal muscle regeneration and reduce fibrosis in aged mice. Twenty‐three‐month‐old mice underwent 10 weeks of aerobic exercise training or received AICAR supplementation. Following the treatment period, the mice were subjected to BaCl_2_ injury at 14 dpi; both aerobic exercise (MICT‐10W) and AICAR supplementation were found to improve the CSA in aged mice (*Figure* *7* *A*). Furthermore, AICAR supplementation demonstrated the ability to enhance satellite cell proliferation and fusion capabilities (*Figure* *7* *B,C*). Masson staining conducted at 14 dpi revealed increased skeletal muscle fibrosis in aged mice compared with the adult group. Notably, AICAR groups showed reduced fibrosis areas (*Figure* *7* *D*). TCF4 IF staining indicated an elevated number of TCF4‐positive cells in aged mice, and both MICT and AICAR significantly attenuated this increase in aged mice (*Figure* *7* *E*). To assess the impact of satellite cell secretion on fibroblast proliferation, media from adult, aged, MICT and AICAR groups were used to culture fibroblasts. Proliferation rates were measured using BrdU staining. The data demonstrated that the growth media derived from aged mice significantly increased fibroblast proliferation, while the media from the MICT and AICAR groups did not exhibit this effect (*Figure* *7* *F*). In summary, these findings suggest that AICAR can emulate the effects of aerobic exercise on skeletal muscle regeneration and fibrosis after skeletal muscle injury in aged mice.

## Discussion

The outcomes of this study shed light on the intricate interplay among aging MuSCs, fibroblast proliferation, skeletal muscle fibrosis and the beneficial effects of aerobic exercise. Our findings reveal an elevation in the secretion of CCN2 from aged MuSCs, contributing to an excessive proliferation of fibroblasts and augmented skeletal muscle fibrosis following injury. Concurrently, the heightened expression of CCN2 inhibits the proliferation and fusion of MuSCs, impairing the regenerative and repair abilities of these cells. Importantly, aerobic exercise demonstrates the capacity to counteract the age‐related increase in CCN2 levels, enhancing the regeneration and repair of skeletal muscle injuries in aging mice. Furthermore, AMPK agonists mimic the effects of exercise, suggesting a potential avenue for therapeutic intervention.

Many investigations have demonstrated that aging negatively impacts the regenerative potential of MuSCs. Factors such as overexpression of P16,^6^ defective autophagy^2^ and diminished cyclin D1 expression^16^ result in impaired activation, compromised mitochondrial fission,^23^ heightened Janus kinase (JAK)/STAT signalling activation^24^ and aberrant epigenetic stress‐induced Hoxa9‐dependent activation,^25^ all contributing to loss of self‐renewal. Physical interactions with the microenvironment and niche signals intricately govern every facet of satellite cell function, ensuring effective regeneration.^26^ The intricate interplay of microenvironmental factors and niche signals is crucial for effective regeneration,^8^, ^12^, ^27^ with emerging evidence suggesting that MuSCs not only respond to niche signals but also actively signal to regulate neighbouring cell types.^28^ MuSCs regulate collagen biosynthesis in fibroblasts through secreted exosomes, which contain miR‐206, in response to hypertrophic stimulation.^17^ However, the effects of aging muscle satellite cells on other cells in their niche remain unknown. In this study, we demonstrated that senescent MuSCs secrete CCN2, which can stimulate fibroblast proliferation, potentially augmenting skeletal muscle fibrosis after injury in aged mice.

CCN2 overexpression in TA skeletal muscle using an adenoviral vector induces muscle damage and regeneration, a fibrotic response and a decrease in skeletal muscle strength.^29^ Conversely, Mdx mice with hemizygous CCN2 deletion or treated with a neutralizing anti‐CCN2 monoclonal antibody (FG‐3019) exhibited improved exercise endurance, better muscle strength, reduced muscle impairment, apoptotic damage and fibrosis.^30^ We also found enhanced CCN2 expression in MuSCs isolated from Mdx mice (data not shown). CCN2 expression is essential for fibroblast proliferation, and in the absence of CCN2, fibroblasts are unresponsive to stimuli like TGF‐β that would normally strongly enhance their proliferation.^31^ Remarkably, in the heart, only CCN2 deletion from activated fibroblasts inhibited the fibrotic remodelling, while deletion from cardiomyocytes (the main source of CCN2 in the heart) had no effects. The secretion of CCN2 by cardiomyocytes is not pro‐fibrotic, while fibroblast‐derived CCN2 can modulate fibrosis.^32^ It is noteworthy that our study revealed that CCN2 overexpression in MuSCs promotes fibroblast proliferation and skeletal muscle fibrosis. Furthermore, this overexpression impairs MuSC differentiation, increases pro‐fibroblast transformation and reduces regeneration ability. CCN2 overexpression did not affect MyoD and MyoG expression but did inhibit the MHC protein expression level. CCN2 impairs the regenerative capacity of MuSCs and increases fibroblast proliferation, collectively leading to defective muscle regeneration and fibrosis. On the other hand, knockdown of CCN2 in aged MuSCs rescues its regeneration ability and decreases skeletal muscle fibrosis after injury.

Compared with young MuSCs, old MuSCs exhibited upregulation of TGF‐β gene sets.^13^, ^16^ Dystrophic muscles are also characterized by TGF‐β signalling and alterations in MuSC fate.^15^ TGF‐β2 repressed the myogenic fate and induced the expression of fibrotic genes such as collagen1 in cultures of murine myoblasts.^15^ Increased Wnt signalling in aged mice leads MuSCs to tend to convert from a myogenic to a fibrogenic lineage.^13^ Importantly, TGF‐β1 and the canonical WNT/β‐catenin pathway mutually stimulate each other via the Smad pathway and non‐Smad pathways such as phosphatidylinositol‐3 kinase (PI3K)/Akt signalling.^33^, ^34^ Given that CCN2 is induced by TGF‐β, increased CCN2 levels in aging MuSCs may be associated with overactivation of the TGF‐β or WNT signalling pathway. Recent studies have shown that AMPK inhibits the TGF‐β signalling pathway.^35^, ^36^ Age‐related MuSC dysfunction was associated with hypophosphorylation of AMPK and its downstream target, p27Kip1. AMPK activation or ectopic expression of a phosphomimetic p27Kip1 mutant was sufficient to suppress in vitro apoptosis, increase proliferation and improve in vivo transplantation efficiency in aged MuSCs.^37^ Importantly, exercise triggers the activation of AMPK, evident through increased Thr172 phosphorylation and subsequent elevated activity, particularly during and immediately post‐exercise.^22^ And AICAR has been one of the most commonly used pharmacological modulators of AMPK activity.^37^ AICAR administration improves MuSC regeneration ability and alleviates the pro‐proliferation effects of aged MuSCs, suggesting that the exercise benefit in MuSCs may occur through improved phosphorylation of AMPK in aged MuSCs, which antagonizes the TGF‐β signalling pathway. However, it is worth noting that we did not assess the AMPK phosphorylation level, and the absence of investigation into the TGF‐β signalling genset represents a limitation in our study. Additionally, apart from fibroblast, FAPs constitute another source of fibroblast contributing to skeletal muscle fibrosis as well.^38^ In the context of FAPs, AMPKα1 knockout in these cells has been shown to promote fibrosis in regenerated muscle tissue. This effect is attributed to the reduction of FAP activation during the initial stage of muscle regeneration, coupled with the suppression of apoptosis during the resolution stage.^39^ Notably, exercise has been demonstrated to enhance skeletal muscle regeneration by promoting senescence in FAPs.^40^ However, the precise mechanism by which exercise reduces aging skeletal muscle fibrosis—whether by directly reducing fibroblast proliferation, inhibiting fibroblast differentiation of FAPs or promoting apoptosis of FAPs—requires further investigation.

In our study, we observed that aerobic exercise significantly reverses the decline in endurance capacity and muscle atrophy in aged mice. Notably, we identified that senescent MuSCs secrete increased CCN2, which, in turn, promotes fibroblast proliferation and impairs the regenerative ability of MuSCs. Intriguingly, exercise demonstrates the capacity to attenuate this deleterious effect. This novel finding has not been reported previously, highlighting a potential avenue for further exploration in understanding the interplay between MuSCs, fibroblasts and the impact of exercise on skeletal muscle fibrosis.

## Conflict of interest statement

No conflicts of interest, financial or otherwise, are declared by the authors.

## Supporting information


**Figure S1.** Assessment of Running Speed and Muscle Histology in Different Age Groups. (a) Maximal running speed of mice across different months. (b) Quadriceps muscle weights were normalized to body weight. (c) H&E staining of quadriceps muscle cross‐sections from Adult, Aged, Aged+Ex‐9M and Aged+Ex‐20M mice and the mean CSA of myofibers was quantified of quadriceps (*n* = 4 mice per group). Scale bar, 100 μm. (d) The number of Pax7 positive cell per section (*n* = 7 per group). (e) Quantification the proportion of γH2AX^+^Ki67^+^ to total Pax7^+^ cells (*n* = 3 for Aged mice, *n* = 5 per other groups). (f) Immunofluorescent staining of TA muscle 5 days after transplantation and quantification results, where Pax7 and Ki‐67 double‐positive cells were quantified and normalized to the total Pax7 positive cell. Data are summarized with mean ± SD; T‐test in panel a; one‐way ANOVA in panel b‐f.
**Figure S2.** MuSC Depletion Causes Skeletal Muscle Regeneration Deficiency. *Pax7* ^CreERT2/+^; *Rosa26* ^DTA/+^ mice were divided into two groups: one group received no tamoxifen injection, while the other underwent one week of tamoxifen injection. (a) At least one week after the final tamoxifen dose, mice were sacrificed, and TA muscle sections were stained for Pax7. Representative images of Pax7 staining and the number of Pax7‐positive cells per image (*n* = 5 per group). Scale bar, 100 μm. (b) Pax7 staining after CTX injury. Scale bar, 100 μm. Data are summarized with mean ± SD; T‐test in panels a.
**Figure S3.** RNA sequencing data of MuSCs from Adult, Aged and Aged+EX‐20M. (a) GSEA enrichment plots for the ECM‐receptor interaction and focal adhesion gene set. NES, normalized enrichment score. (b) Heat map of genes in ECM‐receptor interaction of MuSCs from Adult, Aged and Aged+Ex‐9M groups.
**Figure S4.** Assessment of MuSCs Purity. The MuSCs isolated from mice and purified by pre‐plating underwent one passage and were then plated onto 30 mm culture dishes. After 72 h, cells were fixed and stained with MyoD. (a) Representative images of MyoD staining and the percentage of MyoD‐positive cells in 18 different MuSC samples (b). White arrows indicate the MyoD negative cell. Scale bar, 100 μm.
