# Supplementary figures and images for: Aerobic exercise suppresses CCN2 secretion from senescent muscle stem cells and boosts muscle regeneration in aged mice
Source: J Cachexia Sarcopenia Muscle. 2024 Jun 26;15(5):1733–49. doi: 10.1002/jcsm.13526 (PMC11446704; doi:10.1002/jcsm.13526)

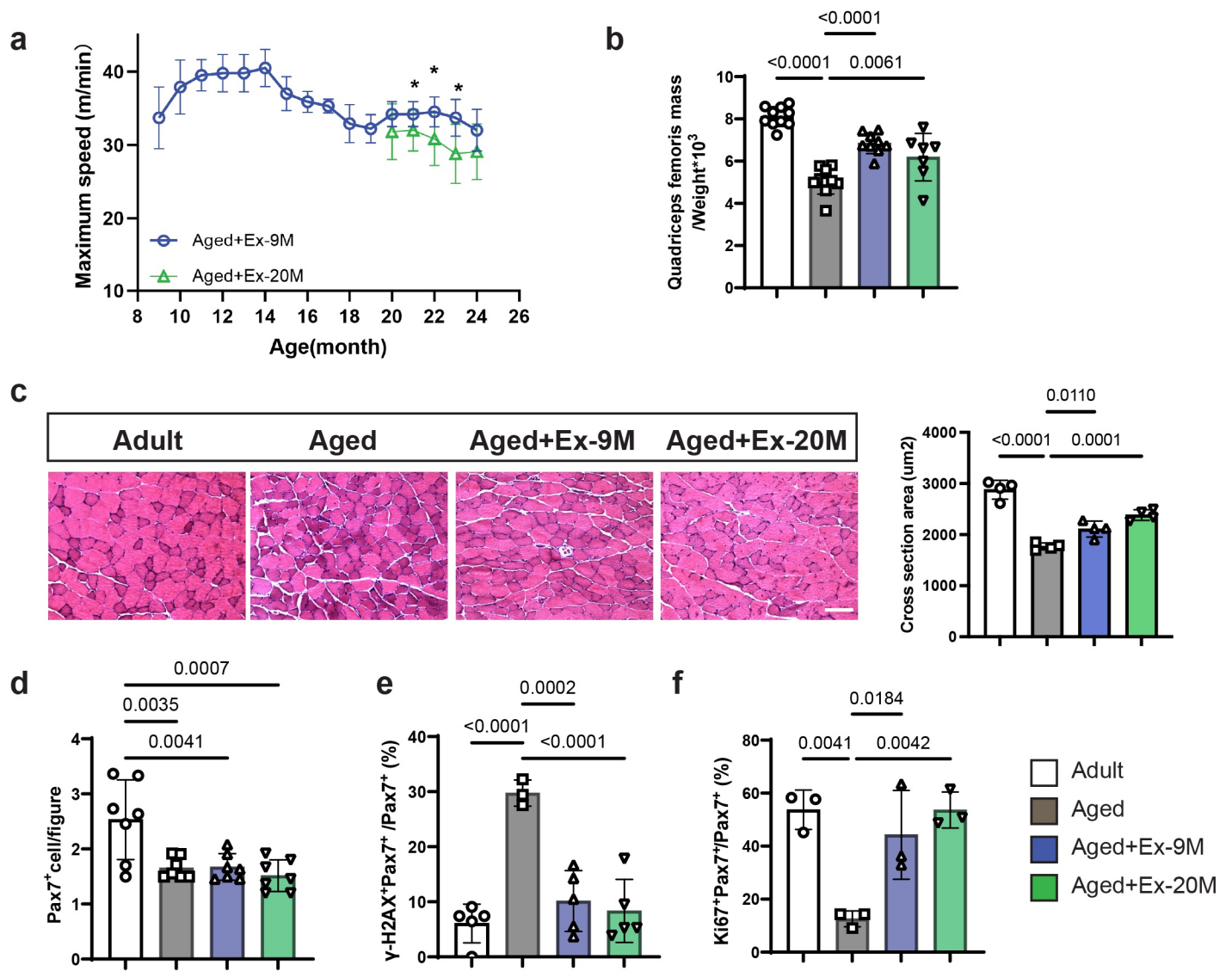

Figure S1

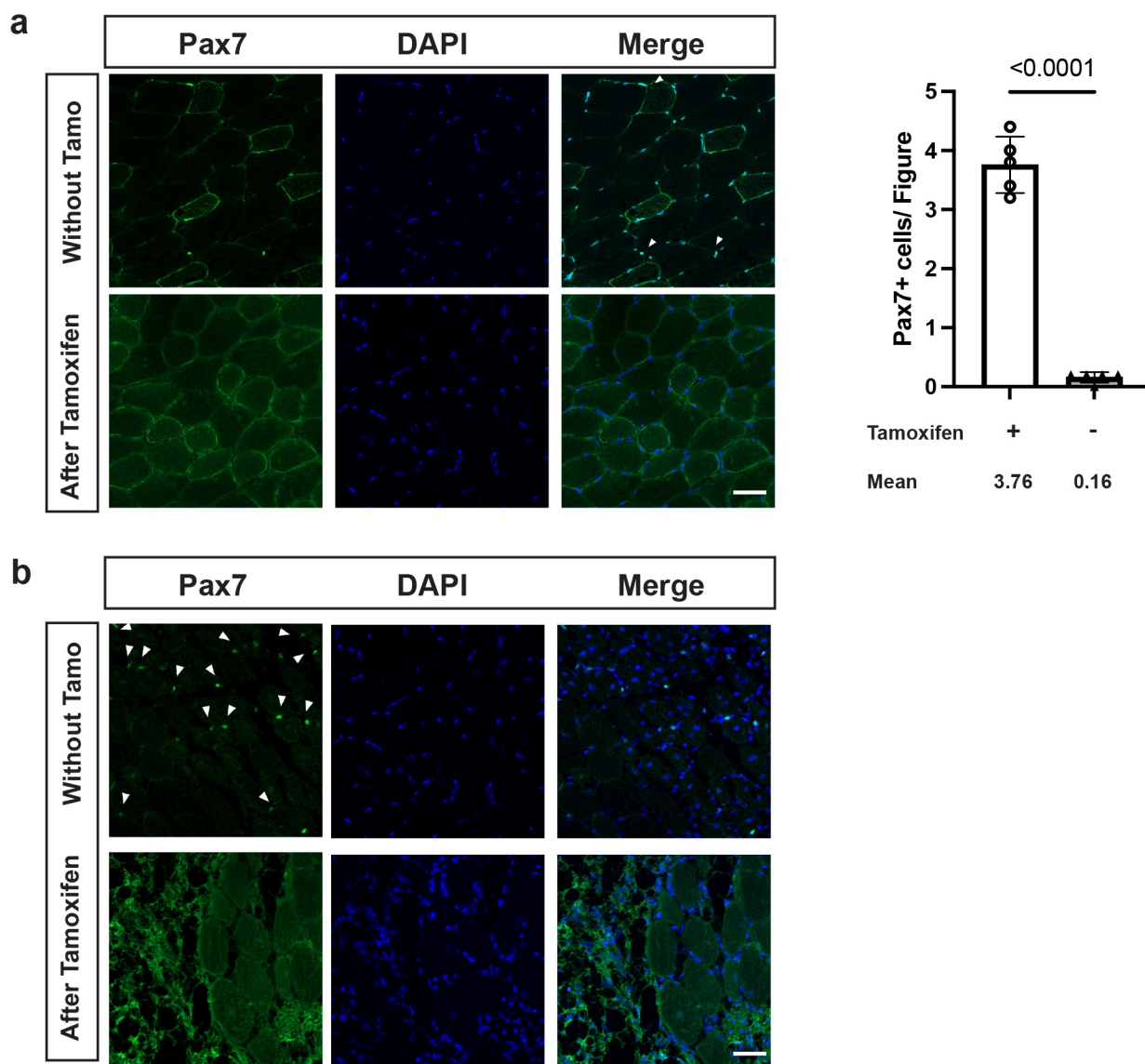

Figure S2

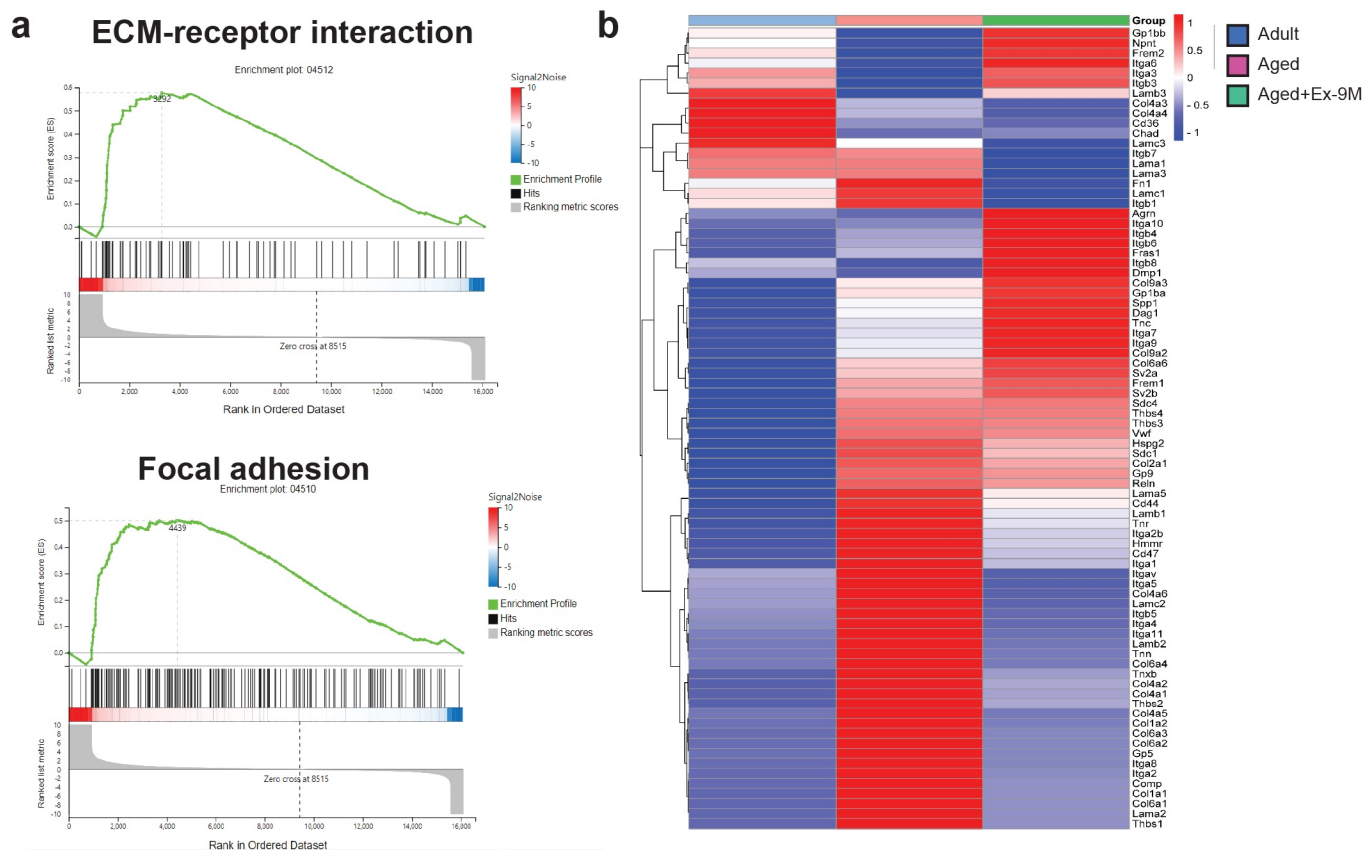

**Figure S3**

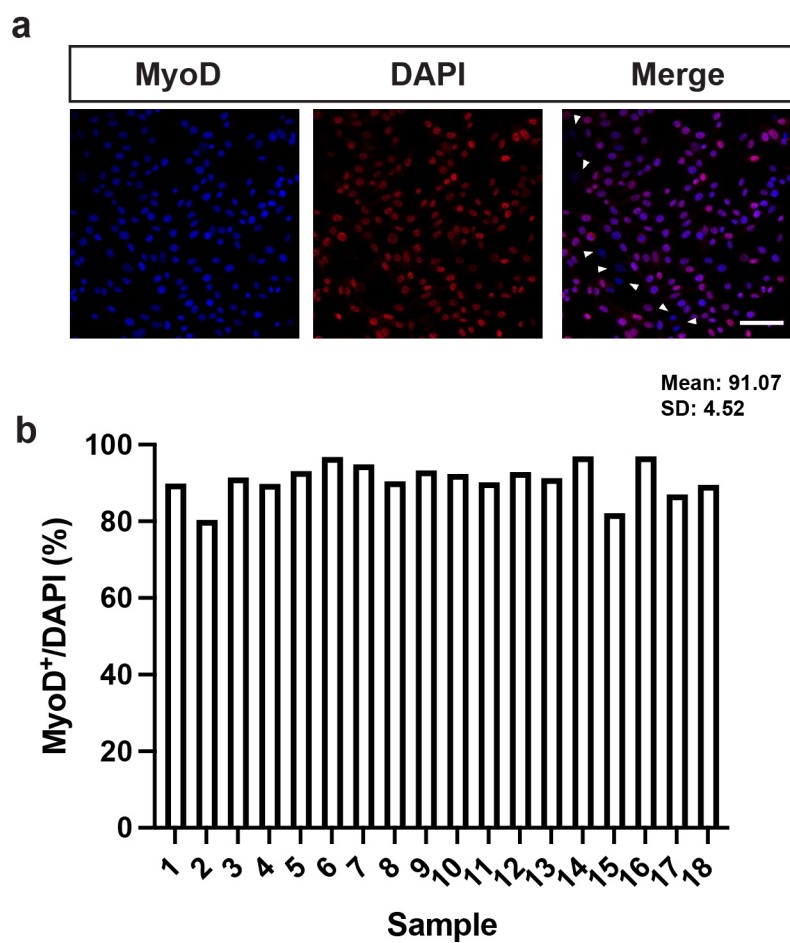

**Figure S4**

Supplement: Supplementary file 1 — Figure S1. Assessment of Running Speed and Muscle Histology in Different Age Groups. (a) Maximal running speed of mice across different months. (b) Quadriceps muscle weights were normalized to body weight. (c) H&E staining of quadriceps muscle cross‐sections from Adult, Aged, Aged+Ex‐9M and Aged+Ex‐20M mice and the mean CSA of myofibers was quantified of quadriceps (n = 4 mice per group). Scale bar, 100 μm. (d) The number of Pax7 positive cell per section (n = 7 per group). (e) Quantification the proportion of γH2AX+Ki67+ to total Pax7+ cells (n = 3 for Aged mice, n = 5 per other groups). (f) Immunofluorescent staining of TA muscle 5 days after transplantation and quantification results, where Pax7 and Ki‐67 double‐positive cells were quantified and normalized to the total Pax7 positive cell. Data are summarized with mean ± SD; T‐test in panel a; one‐way ANOVA in panel b‐f. Figure S2. MuSC Depletion Causes Skeletal Muscle Regeneration Deficiency. Pax7 CreERT2/+; Rosa26 DTA/+ mice were divided into two groups: one group received no tamoxifen injection, while the other underwent one week of tamoxifen injection. (a) At least one week after the final tamoxifen dose, mice were sacrificed, and TA muscle sections were stained for Pax7. Representative images of Pax7 staining and the number of Pax7‐positive cells per image (n = 5 per group). Scale bar, 100 μm. (b) Pax7 staining after CTX injury. Scale bar, 100 μm. Data are summarized with mean ± SD; T‐test in panels a. Figure S3. RNA sequencing data of MuSCs from Adult, Aged and Aged+EX‐20M. (a) GSEA enrichment plots for the ECM‐receptor interaction and focal adhesion gene set. NES, normalized enrichment score. (b) Heat map of genes in ECM‐receptor interaction of MuSCs from Adult, Aged and Aged+Ex‐9M groups. Figure S4. Assessment of MuSCs Purity. The MuSCs isolated from mice and purified by pre‐plating underwent one passage and were then plated onto 30 mm culture dishes. After 72 h, cells were fixed and stained with MyoD. (a [file JCSM-15-1733-s001.pdf]
